# N6-Methyladenosine and 5-Methylcytosine Epitranscriptomic Regulation of Male Reproduction and Fertility

**DOI:** 10.3390/biology15191678

**Published:** 2026-09-22

**Authors:** Sana M. Salih, Ayman Al-Hendy, Qiwei Yang

**Affiliations:** 1 Reproductive Endocrinology and Infertility Section, Department of Obstetrics and Gynecology, University of Chicago, Chicago, IL 60637, USA; sana.salih@bsd.uchicago.edu; 2 Department of Obstetrics and Gynecology, University of Chicago, Chicago, IL 60637, USA; aalhendy@bsd.uchicago.edu; 3 Center for Biotechnology, Khalifa University, Abu Dhabi P.O. Box 127788, United Arab Emirates; 4 Department of Medical Sciences, Khalifa University, Abu Dhabi P.O. Box 127788, United Arab Emirates; 5 Department of Obstetrics and Gynecology, Sheik Shakhbout Medical City, Abu Dhabi P.O. Box 127788, United Arab Emirates

**Keywords:** epitranscriptomics, RNA modifications, m^6^A, m^5^C, male infertility, spermatogenesis, sperm function, germ cells, fertility biomarkers, reproductive epigenetics

## Abstract

Functional spermatozoa production depends on spermatogenesis, a complex developmental process that requires precise regulation of gene expression at multiple levels. Although considerable progress has been made in understanding the transcriptional and epigenetic mechanisms governing germ cell development, post-transcriptional regulation mediated by RNA modifications has recently emerged as an important component of male fertility regulation. Epitranscriptomics, the study of chemical RNA modifications and their associated regulatory proteins, has revealed diverse roles for RNA modifications in spermatogenesis, sperm maturation, fertilization, and early embryonic development. Among more than 170 RNA modifications identified to date, N^6^-methyladenosine (m^6^A) and 5-methylcytosine (m^5^C) are well characterized in the male reproductive system. Distinct groups of regulatory proteins function to deposit, remove, and recognize these modifications, thereby controlling RNA fate and metabolism. Functional studies in genetic knockout animal models show that disrupting key epitranscriptomic regulators contributes to abnormal germ cell development at multiple developmental stages. Notably, adverse environmental exposure reprograms the germ cell epitranscriptome, leading to abnormal spermatogenesis and male infertility. In light of recent advances, this narrative review summarizes current progress in male fertility, with particular emphasis on m^6^A and m^5^C RNA modifications. Finally, it outlines future perspectives on RNA modification studies in male fertility. Deeper mechanistic insights into male infertility can contribute to the development of new targeted therapies.

## Simple Summary

Male infertility has a significant impact on millions of men worldwide and is linked to defects in sperm production, maturation, or function. Although DNA and chromatin regulation have been extensively studied previously, recent research shows that chemical modifications of RNA also play critical roles in male fertility. These RNA modifications, collectively known as the epitranscriptome, regulate processing, translation, and degradation of RNA molecules without changing their underlying sequence. Emerging evidence demonstrates that these RNA modifications regulate key processes during spermatogenesis, including spermatogonial stem cell maintenance, meiosis, spermiogenesis, spermatid development, and sperm function. Abnormal alterations in RNA modification pathways have been associated with poor sperm quality and male infertility. This narrative review summarizes the current understanding of RNA modifications, with a particular focus on m^6^A and m^5^C in male reproductive biology. We discuss and highlight their roles in pathological reprogramming of spermatogenesis and underscore their potential as diagnostic biomarkers and therapeutic targets for the management of male infertility.

## 1. Introduction

### 1.1. Overview of Spermatogenesis in Male Fertility

In males, successful production, maturation, and delivery of functional spermatozoa capable of fertilizing an oocyte and supporting normal embryonic development occur via the process of spermatogenesis [1,2]. Spermatogenesis is a highly regulated and precisely controlled developmental process that occurs within the convoluted seminiferous tubules of the testes [1,3]. The stem cell component of the testis is known as spermatogonial stem cells (SSCs). SSCs proliferate and self-renew to maintain the germ cell pool while generating differentiating spermatogonia that commit to meiosis [1]. Primary spermatocytes undergo homologous recombination and chromosomal segregation to produce haploid round spermatids [1,4]. The sperm then undergo nuclear condensation, acrosome formation, and cytoskeletal remodeling. They also undergo mitochondrial sheath organization and flagellar development in a process known as spermiogenesis, ultimately generating mature spermatozoa [5,6]. After spermatogenesis, sperm must undergo further post-testicular cellular and molecular maturation and functional activation, including capacitation and the acrosomal reaction (AR), to ultimately acquire the ability to combine with an oocyte and complete fertilization [7,8]. Proper germ cell proliferation, differentiation, meiosis, sperm maturation, and competence are important for male fertility.

Somatic cells play a key role in spermatogenesis. Sertoli cells can provide structural and nutritional support and form the blood-testis barrier, protecting developing sperm from the body’s immune system. Leydig cells, on the other hand, regulate spermatogenesis through the synthesis and secretion of testosterone under the control of the hypothalamic-pituitary-gonadal (HPG) axis [1,9,10].

Regulation of male fertility includes transcriptional, post-transcriptional, and translational regulation to ensure proper germ cell proliferation and differentiation [11,12]. Among these mechanisms, post-transcriptional RNA regulation is critically important because transcription is largely silenced during late spermiogenesis, requiring germ cells to maintain appropriate RNA levels and protein production [11,12]. Many studies show that modified RNA transcripts, coordinated with numerous regulatory factors, are essential for maintaining sperm quality, motility, genomic integrity, and fertilization competence [13,14]. Disruption of these regulatory mechanisms can lead to impaired spermatogenesis and male infertility [15,16].

### 1.2. Post-Transcriptional Regulation in Male Germ Cell Development

Spermatogenesis is one of the most complex differentiation systems in mammals and serves as a critical model for understanding reproductive biology, stem cell regulation, germ-somatic cell interactions, and the molecular and epigenetic basis of male infertility [1,2]. Male germ cell development includes multiple processes. SSC maintenance, meiotic divisions, and terminal differentiation require tightly coordinated gene expression. During specific stages of this developmental window, germ cells exhibit markedly reduced transcriptional activity. Accordingly, protein production relies largely on post-transcriptional mechanisms rather than on the synthesis of new transcripts [1,11,17]. To maintain developmental progression under these conditions, germ cells use a broad range of RNA-dependent regulatory processes that control RNA function and fate. RNA modification regulators, RNA-binding proteins, alternative splicing regulators, small non-coding RNAs, translational repressors, and specialized ribonucleoprotein (RNP) complexes can modulate gene expression in the sperm after transcription [18,19].

One well-characterized example of RNA modifications in male germ cells is the chromatoid body, a specialized cytoplasmic RNP granule that supports RNA storage, quality control, degradation, and small RNA-mediated gene regulation. Comparable RNA granules, including processing bodies and maternal messenger ribonucleoprotein complexes, perform related functions during oocyte maturation and the earliest stages of embryonic development [20,21].

These post-transcriptional regulatory mechanisms govern virtually every stage of germ cell development. Among them, RNA modification-regulated mechanisms have attracted significant attention. Functional studies using genetic knockout mouse models demonstrate that disrupting these RNA regulatory networks, which comprise diverse RNA modification regulators, is associated with infertility, defective gametogenesis, impaired embryonic development, and inherited reproductive disorders. Consequently, post-transcriptional regulation represents a fundamental layer of gene control that links the epitranscriptome landscape to functional cellular outcomes during reproduction [22,23,24].

### 1.3. RNA Epitranscriptomic Modifications and Spermatogenesis

Epitranscriptomics encompasses a wide range of chemical modifications to both coding and non-coding RNAs that do not alter the underlying nucleotide sequence [25,26]. More than 170 RNA modifications have been identified across diverse RNA species, including messenger RNA (mRNA), transfer RNA (tRNA), ribosomal RNA (rRNA), microRNA (miRNA), long non-coding RNA (lncRNA), and circular RNA (circRNA) [27,28]. Among the best-characterized modifications are N6-methyladenosine (m^6^A), 5-methylcytosine (m^5^C), pseudouridine (Ψ), N1-methyladenosine (m^1^A), N7-methylguanosine (m^7^G), and adenosine-to-inosine (A-to-I) RNA editing [29,30].

Epitranscriptomic modifications are mediated by specific regulatory proteins referred to as “writers,” “erasers,” and “readers” [25,26]. The writers’ group of regulatory proteins installs specific RNA modifications. The erasers group of regulatory proteins reverses selected modifications. The readers group of regulatory proteins recognizes modified nucleotides, thereby influencing RNA processing and biological function [25,29]. Through the coordinated actions of these proteins, particularly in the m^6^A and m^5^C pathways, RNA modifications regulate RNA splicing, stability, localization, translation, and degradation, thereby providing an additional level of post-transcriptional control over gene expression (Figure 1) [25,31,32]. Because many RNA modifications are reversible and responsive to cellular signals, they provide a highly adaptable mechanism for controlling biological processes [31]. Many studies show that disruption of epitranscriptomic regulation contributes to numerous human diseases, including cancer, cardiovascular disease, neurological disorders, metabolic dysfunction, and infertility [33,34,35,36]. This regulatory system is especially important in reproductive tissues, where transcription is often developmentally restricted, and RNA-based mechanisms play a dominant role in controlling gene expression. Accordingly, RNA modifications have emerged as critical regulators of germ cell development, gametogenesis, fertilization, and early embryonic development [13,14].

### 1.4. Search Methodology

A literature search was conducted in July 2026 to identify articles on RNA modifications, with particular emphasis on m^6^A and m^5^C in male reproduction and fertility. No publication date filter was applied. In PubMed, we used the keywords “RNA Modifications”, “N6-methyladenosine”, “m^6^A”, “5-methylcytosine”, and “m^5^C”. We identified all references that included these keywords in combination with male reproduction using the search terms “male reproduction”, “spermatogenesis”, “sperm”, “testis”, “male fertility”, “infertility”, “spermatogonial stem cells”, “sperm capacitation”, “acrosome reaction”, “zona pellucida”, “oolemma”, “polyspermy block”, “sperm-egg membrane fusion”, and “fertilization”. We also included relevant review articles to provide broader conceptual context. We identified the literature summarized in the tables using the same search strategy and selected articles based on their relevance to the original articles, the experimental evidence they provide, and their contribution to the specific topic addressed in each table. Because this manuscript is a narrative review, we based literature selection on scientific relevance and scope rather than a formal systematic review or PRISMA protocol.

Although several reviews have examined RNA modifications and epitranscriptomic regulation in reproductive biology, this review focuses specifically on m^6^A and m^5^C in male reproduction and fertility. Rather than offering a broad overview of RNA modifications, we synthesize current evidence on the molecular regulators of m^6^A and m^5^C, including writers, erasers, and readers, and on their target RNAs, downstream pathways, and functional consequences in SSC maintenance, meiosis, spermiogenesis, sperm maturation, and male fertility. We also highlight recent advances in m^6^A and m^5^C detection technologies, including single-nucleotide-resolution approaches and direct RNA sequencing, and discuss their current limitations. In addition, we discuss the emerging therapeutic potential of targeting RNA modification pathways while addressing challenges related to specificity, off-target effects, delivery, safety, and potential effects on the germline. We highlight the knowledge gap in defining the role of RNA modifications in acquiring sperm fertilization competence. By integrating mechanistic, technological, and translational perspectives, this review provides an updated framework for understanding how m^6^A and m^5^C contribute to male reproductive biology and identifies key knowledge gaps and directions for future research.

## 2. Sperm Development and Spermatogenesis

Spermatogenesis is the process by which spermatogonial stem cells (SSCs) proliferate and differentiate to generate mature spermatozoa capable of fertilizing oocytes. Spermatogenesis comprises three distinct sequential phases: mitotic proliferation of spermatogonia, meiotic division of spermatocytes, and post-meiotic differentiation of spermatids, commonly referred to as spermiogenesis. Spermatogenesis: The Commitment to Meiosis [1,37,38]. Spermatogenesis is initiated by the proliferation of SSCs located along the basement membrane of the seminiferous epithelium [39,40,41]. After several rounds of proliferation, spermatogonia differentiate and enter meiosis to form primary spermatocytes. The transition from mitosis to meiosis requires extensive changes in both transcriptional and post-transcriptional gene regulation to establish the meiotic program [1,42]. Diploid spermatocytes undergo reduction division via meiosis to give rise to haploid germ cells through a series of tightly regulated events. During prophase I of meiosis, spermatocytes pair homologous chromosomes, undergo synapsis, and exchange genetic material through recombination to generate round spermatids with a haploid chromosome number [43,44,45]. Disruption of meiotic regulation can lead to chromosome missegregation, germ cell loss, meiotic arrest, and ultimately infertility [43,44,45]. Sperm undergo post-meiotic transformation, changing their shape from a rounded, sedentary cell to a motile, spindle-shaped cell that is characteristic of a functional mature spermatozoon [5,46]. During this transformation, sperm acquire an acrosome, undergo cytoskeletal remodeling, assemble the mitochondrial sheath and flagellum, and undergo extensive nuclear condensation driven by the replacement of histones with protamines [46,47,48]. In human sperm, approximately 90% of histones are replaced by protamines. Protamine is a small, highly basic protein that promotes the tight compaction of sperm DNA, thereby protecting paternal genetic material from physical and oxidative damage and supporting proper sperm function and motility [6].

Normal spermatogenesis depends on continuous communication between germ cells and surrounding somatic cells. Sertoli cells establish the structural framework of the seminiferous epithelium, form the blood-testis barrier, and provide nutrients, growth factors, and immunological protection required for germ cell development [1,49,50]. Leydig cells reside in the interstitial compartment and produce testosterone. Testosterone acts both locally, at very high concentrations, to facilitate meiotic progression and spermiogenesis in the testis, and systemically to maintain male reproductive function [51,52]. Bidirectional signaling pathways between Sertoli and Leydig cells are critical for coordinating germ cell proliferation and differentiation.

Like oocytes, sperm transcriptional activity declines progressively and becomes largely silent during meiosis. Developing spermatids rely on transcripts synthesized earlier in development, which must be stored, processed, and translated at precisely defined time points [11,18,53]. Many germ cell transcripts are synthesized well before translation, making RNA storage, stabilization, localization, and translational regulation indispensable for normal development [11,18,53]. Sperm contain specialized ribonucleoprotein complexes and cytoplasmic RNA granules, such as the chromatoid body, that regulate RNA surveillance, processing, and degradation of small RNAs during post-meiotic stages [21,54,55]. These RNA regulatory centers enable developing germ cells to maintain coordinated gene expression despite prolonged transcriptional inactivity.

Evidence accumulated over the past decade has further demonstrated that RNA regulatory pathways contribute to virtually every stage of spermatogenesis, from SSC maintenance and meiotic progression to spermatid differentiation and sperm maturation [11,53,56]. Among these pathways, epitranscriptomic modifications are key regulators of RNA fate and function. RNA modifications dynamically influence transcript stability, splicing, localization, translation, and degradation, thereby shaping germ cell development [25,27,33,57,58]. In this context, understanding how specific RNA modifications contribute to spermatogenesis and male fertility is warranted.

Spermatogenesis relies heavily on post-transcriptional control and is uniquely suited to dissecting RNA-based regulatory mechanisms, as it features prolonged transcriptional quiescence and temporally regulated RNA metabolism. A deeper understanding of RNA regulation in these pathways will not only advance our knowledge of male germ cell biology but also facilitate the identification of molecular mechanisms underlying male infertility and reveal new opportunities for medical intervention (Figure 2).

## 3. m^6^A and m^5^C RNA Modifications in Male Germ Cells: Roles in Spermatogenesis and Sperm Function

### 3.1. Physiological Roles of 5-Methylcytosine (m^5^C) in Spermatogenesis and Testicular Function

The formation of a bipotential gonad initiates development of the male reproductive system. The Y-chromosome–encoded SRY gene triggers testis differentiation [59]. SSCs arise from primordial germ cells (PGCs) that migrate to the testes during development. These SSCs maintain self-renewal and differentiate into mature sperm. Evidence from female reproductive biology suggests that RNA modification is also important for germline function. Alterations in RNA modification pathways have been linked to ovarian insufficiency, abnormal oocyte maturation, and defects in early embryonic development [32,60,61,62]. Together, these findings support the possibility that m^5^C-dependent RNA regulation is an evolutionarily conserved mechanism involved in male reproductive function.

Among the known m^5^C regulators, NSUN7 has attracted considerable attention because of its highly restricted expression in the testis and its indispensable role in male fertility. NSUN7 is predominantly expressed in elongated spermatids during flagellar formation and associates with a distinct set of RNAs enriched in these cells. This spatial and temporal expression pattern and interaction suggest that NSUN7 regulates transcripts required for the final stages of sperm differentiation rather than broadly affecting global RNA metabolism. Consistent with this view, mice lacking *Nsun7* are infertile and exhibit severe defects in sperm motility [63]. Mechanistic studies have shown that loss of *Nsun7* increases the abundance of several NSUN7-associated transcripts, indicating that the protein helps maintain appropriate RNA homeostasis during spermiogenesis. Deficiency of NSUN7 also disrupts sperm structure, compromising flagellar function and resulting in reduced sperm motility and male infertility [64,65].

Interestingly, mutating the canonical catalytic cysteine of NSUN7 does not impair male fertility or alter flagellar architecture, suggesting that its biological function extends beyond its predicted methyltransferase activity [66]. This observation is supported by biochemical and structural studies showing that NSUN7 lacks detectable RNA m^5^C methyltransferase activity. Unlike catalytically active members of the NSUN family, NSUN7’s IV motif contains a leucine residue rather than the conserved aspartate required for S-adenosylmethionine (SAM) binding and catalysis [64]. Consequently, deletion of *Nsun7* does not produce measurable changes in global or transcriptome-wide m^5^C levels. Instead, it alters the expression of genes involved in ciliary assembly and cytoskeletal organization, including *Cfap20*, *Cfap45*, *Cfap52*, *Wdr35*, *Tuba3a*, *and Tuba3b*, resulting in axonemal abnormalities, reduced sperm motility, and male infertility [64]. These findings support the view that NSUN7 functions through a non-canonical, methyltransferase-independent mechanism, primarily as an RNA-associated regulatory and structural protein rather than a conventional m^5^C methyltransferase.

Additional components of the m^5^C regulatory machinery are required for normal spermatogenesis. NSUN2, which localizes to the chromatoid body of germ cells, catalyzes tRNA methylation and contributes to post-transcriptional RNA processing. Loss of *Nsun2* disrupts meiotic progression at the pachytene stage, reduces germ cell differentiation, and ultimately causes male infertility [65]. Another important factor is YBX1, an RNA-binding protein that functions as an m^5^C reader. Although meiosis proceeds normally in the absence of YBX1, mutant animals exhibit fewer elongated spermatids, lower sperm counts, impaired sperm motility, and reduced fertilization capacity, highlighting a critical role in the post-meiotic phase of spermatogenesis [67].

TSGA10, a testis-specific protein, has been shown to interact with both NSUN2 and GRP78 to ensure proper mitochondrial sheath formation in spermatozoa. Consistent with the *Nsun2* knockout mouse model, *Tsga10* deficiency leads to male infertility, disordered mitochondrial sheath formation, and reduced sperm motility, highlighting a connection among structural integrity, post-transcriptional regulation, and RNA modification pathways in spermatogenesis [68].

Studies in livestock and other vertebrate species further suggest that these regulatory mechanisms are evolutionarily conserved. Direct RNA sequencing of testes from prepubertal, pubertal, and postpubertal bulls identified more than 5000 genes whose expression changed during testicular maturation. Many of these genes displayed dynamic alternative splicing events, including exon skipping and alternative splice-site usage, that were associated with both m^5^C and m^6^A RNA modifications. In particular, m^5^C-associated splicing promoted isoform-specific expression of spermatogenesis-related genes, such as MAEL, which support normal germ cell development and testicular maturation [69]. Genome-wide transcriptomic analyses of rooster testes showed the expression of endogenous RNA (ceRNA) pathways, including MAPK, TGF-β, and mTOR, and the involvement of YBX1 in these regulatory networks, suggesting that m^5^C-dependent mechanisms aid sperm formation and reproductive function in avian species. In medaka fish, paternal exposure to methotrexate alters the small RNA composition of sperm and increases m^5^C modification via DNMT2. Injection of these sperm RNAs into fertilized eggs results in developmental abnormalities in the offspring, providing experimental evidence that RNA modifications can contribute to paternal epigenetic inheritance [70,71].

The translational relevance of m^5^C regulation is also emerging. In a busulfan-induced mouse model of oligoasthenospermia, treatment with 5-methylcytidine restored m^5^C levels and improved several indicators of reproductive function, including testicular morphology, sperm concentration and motility, cytoskeletal organization, ciliary structure, and hormonal balance. The treatment also reduced oxidative stress and promoted recovery of spermatogenesis, suggesting that modulating m^5^C pathways may have therapeutic value in selected forms of male infertility [72].

Current evidence indicates that m^5^C-associated regulatory proteins, including NSUN2, NSUN7, YBX1, and DNMT2, participate in multiple aspects of spermatogenesis, ranging from RNA metabolism and germ cell differentiation to sperm structural integrity, motility, and epigenetic inheritance. Rather than acting through a single mechanism, these proteins appear to function within interconnected post-transcriptional regulatory networks essential for normal male reproductive physiology (Table 1).

### 3.2. m^6^A Regulation Throughout Spermatogenesis

#### 3.2.1. m^6^A Regulation of Spermatogonial Stem Cell Maintenance and Differentiation

Accumulating evidence suggests that m^6^A contributes to SSC self-renewal and differentiation. In germ cells, the METTL3–METTL14 methyltransferase complex establishes stage-specific m^6^A patterns that support the translational programs required for progression along the spermatogenic lineage. Conditional deletion of either *Mettl3* or *Mettl14* in mouse germ cells disrupts these regulatory programs, leading to SSC depletion, impaired translation of key regulatory transcripts, and severe defects in spermatogenesis that culminate in infertility [13]. The surrounding somatic niche is equally important for maintaining SSC homeostasis. In Sertoli cells, WTAP, an essential component of the m^6^A methyltransferase complex, is required for normal niche function. Loss of *Wtap* alters m^6^A-dependent RNA splicing and the post-transcriptional regulation of transcripts that support germ cells. As a consequence, SSCs gradually become depleted, eventually leading to the complete failure of spermatogenesis [73]. These findings indicate that successful SSC maintenance depends on coordinated m^6^A regulation within both germ cells and their supporting somatic environment (Figure 3).

#### 3.2.2. m^6^A-Mediated Control of Meiotic Initiation and Progression

The transition from mitotic proliferation to meiosis is one of the most tightly regulated steps in spermatogenesis, and m^6^A signaling plays a key role in this developmental switch. A key component of this pathway is the m^6^A reader YTHDC2, which promotes degradation of transcripts associated with the mitotic program and facilitates expression of genes required for meiosis. Mice lacking *Ythdc2* fail to properly silence mitotic regulators such as Ccna2, resulting in defective meiotic entry, disruption of germ cell identity, meiotic arrest, and infertility [74,75].

Subsequent studies have shown that the functions of YTHDC2 extend beyond its canonical role as an m^6^A reader. In addition to recognizing methylated RNA, YTHDC2 contributes more broadly to RNA turnover during meiotic prophase. Deficiency of this protein causes abnormalities in microtubule organization, defective telomere clustering, and extensive apoptosis at the pachytene stage [76]. Rather than acting through a single mechanism, YTHDC2 appears to integrate multiple aspects of RNA metabolism to ensure orderly meiotic progression (Table 2) (Figure 3).

#### 3.2.3. Roles of m6A in Spermiogenesis and Sperm Maturation

Developing spermatids rely heavily on post-transcriptional regulation of pre-existing RNA because transcription is silenced during the later stages of spermiogenesis. Under these conditions, m^6^A modification provides an important mechanism for coordinating RNA metabolism and protein synthesis. One of the best-characterized regulators is the m^6^A demethylase ALKBH5, which contributes to RNA processing and the maintenance of transcript diversity during the later stages of germ cell development. Deletion of *Alkbh5* increases global m^6^A abundance, alters alternative splicing patterns, and promotes the generation of unstable transcript isoforms that are rapidly degraded. These molecular changes are accompanied by defective sperm development, abnormal morphology, reduced motility, and phenotypes resembling oligoasthenoteratozoospermia [77,78]. Genome-wide MeRIP-seq and RNA-seq analyses of pachytene spermatocytes and round spermatids from *Alkbh5* knockout mice revealed extensive remodeling of the m^6^A epitranscriptome during late spermatogenesis. Differentially methylated transcripts were enriched within coding sequences and stop codons containing the conserved GGAC motif and were associated with spermatogenesis-related pathways. Integrated methylome-transcriptome analyses further demonstrated that ALKBH5-mediated demethylation provides new insights into the molecular mechanisms of m6A modification in germ cell development and spermatogenesis [79]. These molecular defects result in abnormal sperm morphology, reduced motility, and oligoasthenoteratozoospermia-like phenotypes.

Another regulator implicated in spermiogenesis is the m^6^A reader IGF2BP3. Mice lacking *Igf2bp3* exhibit abnormal sperm morphology together with reduced sperm number and motility, indicating an important role in sperm maturation [80]. Mechanistic studies suggest that IGF2BP3 helps coordinate the timing of protein synthesis during histone-to-protamine replacement by repressing translation of Dot1l, a histone methyltransferase, and Hdac11, a histone deacetylase. This regulation depends on both m^6^A recognition and interaction with the RNA-binding protein YBX2 [80]. Loss of *Igf2bp3* and excessive production of DOT1L and HDAC11 disrupt chromatin remodeling and compromise sperm chromatin organization, leading to inefficient removal of histones and improper deposition of protamine [80]. The abnormal retention of histones in mature sperm may interfere with protamine binding to DNA, thereby impairing proper sperm DNA compaction and chromatin organization.

The influence of m^6^A extends beyond messenger RNAs. Recent studies have shown that this modification also regulates the translation of non-coding RNAs during late spermatogenesis. m^6^A promotes both the biogenesis and cap-independent translation of circular RNAs (circRNAs), many of which are enriched near translational start and stop codons. Translation of these modified circRNAs yields functional peptides that help sustain protein synthesis in transcriptionally quiescent spermatids, thereby supporting elongation, maturation, and the acquisition of sperm function [81]. These findings broaden the scope of m^6^A-dependent regulation by demonstrating that both coding and non-coding RNAs contribute to the molecular events underlying spermiogenesis (Table 2) (Figure 3).

#### 3.2.4. Contributions of Sertoli and Leydig Cell Epitranscriptomics

Epitranscriptomic regulation in the testis extends beyond germ cells. Supporting somatic cells also rely on m^6^A-dependent mechanisms that influence the local environment required for spermatogenesis. In Sertoli cells, WTAP is an essential component of the m^6^A methyltransferase complex that maintains the SSC niche. Conditional deletion of *Wtap* in Sertoli cells of *Wtap*-sKO mice disrupts RNA splicing and post-transcriptional regulation of gene expression, leading to progressive loss of undifferentiated spermatogonia and male infertility [73]. Leydig cells provide another example of cell type-specific m^6^A regulation. A study reported that alterations in the expression of *METTL14* and *ALKBH5* reduce overall m^6^A methylation and enhance autophagy in Leydig cells. This m^6^A modification reduced AMPK activity, thereby inhibiting autophagy. Notably, human chorionic gonadotropin treatment also upregulated ALKBH5 and downregulated METTL14 [94]. These studies uncover a regulatory mechanism in somatic cells that contributes to normal spermatogenesis.

#### 3.2.5. Roles of m^6^A in Epididymal Sperm Maturation and Storage

Sperm exit the testes and travel through the epididymis, where they mature and acquire motility and the ability to fertilize eggs. Sperm are then stored in the cauda epididymis until ejaculation or replacement by fresh sperm. Although mature epididymal sperm are generally considered transcriptionally and translationally quiescent due to nuclear condensation and the shedding of cytoplasmic machinery, their RNA cargo is not static. During epididymal transit, sperm undergo post-transcriptional remodeling of their RNA cargo through interactions with the epididymal environment. These processes are supported by multiple sources, including the remnant cytoplasmic droplet, secreted epididymal proteins, and extracellular vesicles known as epididymosomes. Recent studies have shown that ejaculated sperm undergo limited translation of select proteins required for motility, capacitation, and survival [100]. Sperm also undergo lipid remodeling, with increased production of phosphatidylcholine and sphingomyelin to enhance membrane integrity and mobility [101]. Additionally, sperm acquire increased levels of tRNA-derived fragments (tsRNAs) and microRNAs [102].

RNA modification signatures regulate sperm translation and transcript stability during epididymal transit [103]. Differential m^6^A expression across epididymal regions indicates that each part of the tube has a unique, specialized role in sperm maturation. Upon exiting the testis, caput sperm exhibited a sudden spike in m^5^C and a decrease in m^6^A [104]. This was followed by a gradual increase in m^6^A as sperm entered the corpus. m^6^A methylation was mapped across the three regions of the male epididymis (the caput, corpus, and cauda) [105]. Distinct regional differences in m^6^A methylation levels and associated regulatory gene expression were identified in the epididymis. The highest m^6^A methylation level was observed in the corpus. This was associated with increased levels of m^6^A readers and writers (*YTHDF2*, *YTHDC1*, *RBM15*, *METTL14*, *WTAP*, *VIRMA*) and decreased levels of m^6^A erasers (*FTO* and *ALKBH5*) in the corpus [105].

Importantly, these observations should not be interpreted as evidence that m^6^A directly regulates de novo translation in mature epididymal sperm. Instead, m^6^A remodeling may influence the stability, persistence, localization, and functional potential of sperm-borne transcripts and small RNAs during epididymal maturation. Thus, changes in m^6^A may serve as a post-transcriptional maturation-associated signature that contributes to the selective retention or turnover of sperm RNAs and may influence sperm function after fertilization. Consistent with this concept, transcriptome-wide m^6^A profiling of sperm from myostatin (MSTN)-edited bulls revealed over 1000 differentially methylated genes, mainly involved in G protein-coupled receptor signaling, membrane organization, and cytoskeletal regulation. Further analysis identified RHOA as a central regulatory hub, with several associated genes. Conventional semen parameters remained unchanged in these bulls despite these epitranscriptomic changes. This suggests that m^6^A remodeling may regulate sperm molecular composition and function without altering standard sperm quality measures. These findings position m^6^A as a potential molecular marker to improve sperm function beyond traditional phenotypic assessments [83].

#### 3.2.6. Sperm Capacitation, Acrosome Reaction, and Oocyte Fertilization

Sperm-oocyte interaction is a highly regulated, evolutionarily conserved process that includes sperm capacitation, penetration of the cumulus granulosa cell (CC) layer, binding to the zona pellucida (ZP), the acrosome reaction (AR), and gamete membrane fusion [106,107,108]. Oocytes are ovulated as cumulus-oocyte complexes. Each cumulus-oocyte complex consists of a mature oocyte, enclosed by a thick, protective outer glycoprotein layer (ZP) and surrounded by a few layers of specialized CC connected to the oocyte by dense gap junctions. Sperm must overcome several barriers, including the CC layer, the ZP, and the oocyte plasma membrane (oolemma), to reach and fertilize the oocyte. Fusion of the sperm and the oocyte triggers a polyspermy block, oocyte activation, and fertilization, culminating in the formation of the male pronucleus and the zygote.

Sperm are chemically attracted to the oocyte by factors produced by the CC and the oviductal epithelium, including DEFB119, CCL20, and progesterone. These factors modulate Ca2+ influx through channels such as the CatSper and TRP channels, thereby guiding sperm motility by regulating intracellular calcium [8,109]. Sperm undergo capacitation and acquire a hyperactivated state as they travel through the female genital tract. Sperm shift from steady swimming to high-amplitude, asymmetric, and nonlinear movement essential for reaching the oocyte [110]. After passing through the CC, capacitated, hyperactivated sperm traverse the extracellular matrix by activating sperm-bound hyaluronidases and proteases, such as SPAM1, HYAL5, Acrosin, and Proteasomes, to digest the hyaluronidase-rich matrix protein of the Cumulus-oocyte complex (COC) [8].

The AR is essential for sperm penetration of the ZP. During the AR, the sperm plasma membrane fuses with the outer acrosomal membrane via SNARE proteins, enabling the acrosomal cap to release potent proteolytic enzymes, such as acrosin, and to expose surface ligands, including the Izumo sperm-egg fusion protein 1 (IZUMO1), on the sperm surface [111,112,113]. Once sperm reach the zona pellucida, they bind to specific glycoprotein receptors within the ZP. Multiple complementary ZP matrix glycoprotein receptors, such as ZP3 and ZP4, bind to specific ligands on the sperm plasma membrane (such as SED1, GalTase, and fertilin complexes) [108]. The specificity and complementarity of these receptors coordinate gamete recognition and are essential for the species-specific sperm-ZP interaction, ensuring that only human sperm can fertilize a human egg [108]. The exact location and triggers of the AR and exocytosis are not well known [109]. While acrosome-reacted sperm have been recovered from oviductal fluid, the cumulus-oophorus, the CC, and the surface of the ZP in mammals, human sperm appear to mostly undergo the AR upon reaching the intact zona surface [8,114]. Adhesion of capacitated sperm to the zona pellucida triggers calcium influx through CatSper and TRP family channels, initiating the AR [8].

Further fusion of the spermatozoan head with the oolemma involves the binding of specific ligands on the sperm head to matching receptors on the oocyte surface, leading to complete integration of the sperm cell into the oocyte cytoplasm [108,113,115]. Specifically, the binding of the IZUMO1 receptor on the spermatozoon head to its ligand, the JUNO protein on the oolemma, mediates gamete fusion [111,116]. CD9, a tetraspanin expressed on the oolemma at the site of the membrane fusion machinery, further facilitates this process [116]. The polyspermy block is essential to prevent polyploid embryos that are incompatible with a healthy pregnancy. It begins after sperm-egg fusion and occurs at the zona pellucida and the oolemma in mammalian eggs. Polyspermy block and egg activation are mediated by the release of the sperm-specific isoform phospholipase C zeta (PLCζ) into the egg cytoplasm, triggering Ca^2+^ oscillations and cortical granule exocytosis, which releases proteases and metalloproteinases such as ovastacin [117]. Ovastacin cleaves and inactivates the N-terminus of ZP2, thereby rendering ZP unable to bind additional sperm [118]. Additionally, shedding of the Juno protein from the oolemma into the perivitelline space renders the oocyte membrane unreceptive to sperm fusion [116,119].

Egg activation and the resumption and completion of oocyte metaphase II are driven by intracellular Ca2+ oscillations, which trigger degradation of early mitotic inhibitor 2 (Emi2) and the downstream inactivation of the MAPK and maturation-promoting factor (MPF) pathways [120]. The oocyte arrested at metaphase II completes meiosis upon activation. It extrudes the second polar body and forms the haploid female pronucleus in preparation for syngamy and zygote formation [121]. Similarly, the sperm sheds its tail and replaces the sperm-specific protamines that package the paternal DNA with maternal histones, thereby facilitating chromatin decondensation and male pronucleus formation. After the nuclear envelopes of both pronuclei break down and the genomes mix, the maternal and paternal chromosome sets align along the metaphase plate of a shared mitotic spindle apparatus to achieve syngamy, thereby completing fertilization and initiating embryonic cleavage [119,121].

Therapeutic advances in fertility treatments recapitulate natural in vivo sperm capacitation, selection, and AR in andrology and embryology laboratories. Sperm capacitation for intrauterine insemination (IUI), in vitro fertilization (IVF), and intracytoplasmic sperm injection (ICSI) is achieved by washing and incubating sperm in albumin (10% Human Serum Albumin) and bicarbonate-enriched culture media to stimulate intracellular calcium influx and artificially activate sperm [8]. Conventional IVF mimics natural fertilization by mixing sperm with the COC in the laboratory. The ICSI procedure, on the other hand, involves manual selection of the best-looking motile sperm, followed by mechanical immobilization by crushing the sperm flagellum to release PLCζ before injecting the sperm into the oocyte [122,123]. PLCζ mutations are associated with male infertility, including recurrent ICSI and fertilization failure, which can be overcome by calcium ionophores and recombinant *PLCZ1* microinjection [122,123].

Recent literature has comprehensively reviewed the pivotal roles of the m^6^A and m^5^C RNA modifications and their regulatory machinery in spermatogenesis, as well as their functions in maternal mRNA degradation, the maternal-to-zygotic transition, and early embryo development [56,109,124,125,126,127]. However, the potential involvement of m^6^A and m^5^C in sperm capacitation, acrosome exocytosis, membrane fusion, and chromatin remodeling warrants further investigation. Limited data suggest that m^6^A and m^5^C are essential for these processes.

Studies have shown that m^5^C is important for paternal epigenetic inheritance. Transgenerational transmission of paternal mental stress- and diet-induced metabolic phenotypes is mediated by sperm small non-coding RNAs (sncRNAs), including tRNA-derived small RNAs (tsRNAs) [128]. tsRNAs upregulate metabolic pathways in the early mouse embryo, leading to glucose intolerance in high-fat-fed mice [128]. This metabolic phenotype was abolished when *Dnmt2* deletion prevented m^5^C and N2-methylguanosine (m^2^G) RNA modifications, or when chemically synthesized unmodified tsRNAs were injected into the zygote. These findings indicate that epitranscriptomic RNA modifications, including m^5^C, enhance the stability and longevity of sperm-derived tsRNAs, thereby mediating paternal epigenetic inheritance [128,129].

#### 3.2.7. Environmental Influences on the Testicular m^6^A Landscape

Multiple studies demonstrate that environmental and metabolic stressors strongly disrupt the testicular m^6^A epitranscriptome. Exposure to endocrine-disrupting chemicals (EDCs) such as DEHP alters global m^6^A levels and modifies the expression of FTO and YTHDC2, leading to enhanced m^6^A modification of the antioxidant regulator Nrf2 mRNA. This suppresses antioxidant signaling, induces oxidative stress, and results in germ cell apoptosis and impaired spermatogenesis. In parallel, another EDC, atrazine, induces widespread remodeling of the testicular m^6^A methylome. m^6^A transcriptome-wide map of an amphibian species, *X. laevis*, demonstrated that atrazine altered the expression profile at 1380 m^6^A modification sites, disrupting key pathways involved in testicular development [90].

Similarly, varicocele-associated oxidative stress promotes IGF2BP2-dependent stabilization of m^6^A-modified *Plod2* transcripts, thereby driving germ cell dysfunction [88]. Targeted demethylation with CRISPR/dCas13b-ALKBH5 can significantly reduce m^6^A levels and *PLOD2* expression, thereby lowering oxidative stress and potentially serving as a novel therapeutic target for male infertility [88].

#### 3.2.8. Clinical Implications of m^6^A Dysregulation in Male Infertility

Aberrant m^6^A regulation is important in human male infertility, as shown by clinical studies. Genetic and epitranscriptomic alterations in m^6^A regulators further support causality. Missense mutations in the eraser protein FTO, identified by sequencing a selected region of the *FTO* gene, are associated with reduced semen quality and defective spermatogenesis [87]. Additionally, abnormally elevated m^6^A levels and increased METTL3 expression were observed in patients with asthenozoospermia and correlated with impaired sperm function and infertility phenotypes [84]. This finding is consistent with a study showing that DEHP exposure increases global m^6^A levels, which are associated with testicular toxicity in an animal model [86].

Importantly, paternal m^6^A dysregulation has been identified in spermatozoa from men in couples in the recurrent pregnancy loss group. MeRIP sequencing analysis revealed altered m^6^A methylation and expression of *SEMA5A*, *MT-ATP6*, *ZNF662*, and *KDM4C* [85]. The important role of these four genes in embryonic development was further characterized in zebrafish embryos using knockdown or overexpression approaches, indicating that their dysregulation disrupts embryogenesis and may contribute to unexplained recurrent pregnancy loss [85]. These findings highlight sperm m^6^A methylation as both a functional regulator and a potential diagnostic biomarker for reproductive outcomes.

## 4. Future Directions

### 4.1. Mapping the Full “Male Germline Epitranscriptome”

Although considerable progress has been made in characterizing the role of RNA modifications, the epitranscriptomic landscape of the male germline remains largely unknown. Spermatogenesis and the subsequent functional maturation of sperm involve continuous transitions among SSCs, spermatocytes, spermatids, and mature spermatozoa, with each stage relying on distinct transcriptional and post-transcriptional regulatory programs. In this context, every developmental stage likely has a characteristic epitranscriptomic profile that influences RNA stability, translation, alternative splicing, and cell differentiation.

High-resolution, cell-type-specific maps of RNA modifications are required to assess male fertility throughout the male reproductive tract, including the testis, epididymis, seminal plasma, and mature sperm. High-throughput sequencing and integrated bioinformatic analyses enable the detection of multiple RNA modifications with increasing sensitivity and spatial resolution. These approaches detect RNA modifications not only in mRNAs but also in other RNA classes, including tRNAs, rRNAs, miRNAs, piRNAs, lncRNAs, circRNAs, and sperm-derived small RNAs, thereby providing a more comprehensive picture of epitranscriptomic alterations in germ cell biology and paternal inheritance. Integrating epitranscriptomic data with transcriptomic, proteomic, and chromatin accessibility datasets will provide a more comprehensive view of RNA regulation during male reproduction. Such integrated resources could also facilitate the discovery of biomarkers associated with male infertility and identify new candidates for therapeutic intervention.

### 4.2. Functional Role of RNA Modifications in Male Fertility

Recent genetic evidence shows that RNA modification regulators play important roles in male fertility. In the context of m^6^A RNA modification, genetic deletion or knockout of components of the m^6^A methyltransferase complex, including METTL3, METTL14, and WTAP, disrupts spermatogenesis and impairs sperm development. Similarly, studies using ALKBH5- and FTO-deficient mouse models have revealed abnormalities in germ cell development and spermatogenesis, further supporting the importance of m^6^A demethylation in male fertility. m^6^A readers also contribute to the regulation of male germ cell development. Loss of *Ythdc2* or *Ythdf2* has been associated with impaired spermatid development, abnormal sperm chromatin remodeling, and defects in the transition from mitosis to meiosis. In addition, IGF2BP3 has been implicated in the regulation of germ cell development, although its specific contribution to spermatogenesis remains to be fully defined. Future studies that identify the individual transcripts regulated by these RNA modification factors and determine how their regulation influences specific developmental stages during spermatogenesis are warranted. In the context of m^5^C RNA modification, accumulating evidence similarly indicates that m^5^C regulators, particularly the RNA methyltransferases of the NSUN family, play important roles in male fertility. Several studies have shown that NSUN7 deficiency disrupts sperm flagellar formation during spermatid maturation, resulting in impaired sperm motility and male infertility [63,64,66]. Similarly, genetic deletion of *Nsun2* causes meiotic arrest at the pachytene stage and disrupts spermatogenesis. Interestingly, TSGA10, which interacts with NSUN2, has also been implicated in male fertility. Loss of *Tsga10* results in male infertility, reduced sperm motility, and abnormal mitochondrial sheath formation. In addition to m^5^C writers, the m^5^C reader YBX1 is involved in male reproductive function. *Ybx1* deficiency has been associated with reduced sperm count and motility, as well as impaired fertilization capacity, ultimately leading to male subfertility. Future studies should extend research to identify the key transcripts modulated by these RNA modification regulators and to determine how their modification-dependent regulation contributes to distinct stages and developmental events during spermatogenesis.

### 4.3. Stress-Induced RNA Modifications as a Potential Regulatory Mechanism in Male Infertility

Recent work has identified an additional layer of RNA regulation that may be relevant to male reproduction under adverse environmental conditions. Under cellular stress, pseudouridine synthase 7 (PUS7), an enzyme that catalyzes Ψ formation in multiple RNA species [130], relocalizes from the nucleus to the cytoplasm and promotes widespread pseudouridylation of messenger RNAs [131]. These findings raise the possibility that environmental stress may alter the localization of other RNA modification regulators, thereby reshaping the m^6^A/m^5^C epitranscriptome in male germ cells or in supporting somatic cells.

Endocrine-disrupting chemicals (EDCs) may perturb physiological stress-response pathways. In a rat model, DEHP can enhance oxidative stress, inhibit the Nrf2-mediated antioxidant pathway, and is associated with increased global levels of m^6^A modification and altered expression of *FTO* and *YTHDC2* [86]. In Xenopus laevis testicular tissue, Atrazine induced changes in multiple m^6^A-related pathways involved in modification, contributing to abnormal testicular development and highlighting an epitranscriptomic mechanism underlying adverse environmental effects [90]. Further studies are needed to identify susceptible populations, establish causal mechanisms, and develop biomarkers and preventive or therapeutic strategies for stress-associated male infertility.

Spermatogenesis is particularly vulnerable to environmental challenges, including heat stress, oxidative injury, inflammation, and disturbances in metal ion homeostasis. Therefore, the epitranscriptomic mechanisms underlying stress-induced responses require in-depth investigation. Determining how these RNA modification pathways respond to cellular stress by analyzing the m^5^C and m^6^A epitranscriptomic landscape may provide insight into the mechanisms that preserve germ cell survival, regulate meiotic progression, support spermatid differentiation, and maintain sperm function under adverse conditions.

### 4.4. Recent Advances in m^6^A and m^5^C Detection Technologies and Limitations

Recent advances in transcriptome-wide detection of RNA modifications have substantially improved the ability to characterize the distribution and dynamics of m^6^A and m^5^C across the transcriptome. Conventional antibody-based approaches, including m^6^A-seq (MeRIP-seq) and m^5^C-RIP-seq, have been widely used to identify modification-enriched regions and have provided important insights into the roles of these modifications in reproductive diseases. However, these methods generally have limited spatial resolution and depend on antibody specificity, making it difficult to determine the precise modified nucleotide and, in some cases, to distinguish closely related modification sites [132].

Recent advances in sequencing technology enable qualitative and quantitative analysis of m^6^A and m^5^C in RNAs at single-nucleotide resolution, including miCLIP-based methods for m^6^A and bisulfite-based or related chemical-conversion approaches for m^5^C [133,134]. These methods allow the identification of m^6^A and m^5^C deposition in vitro and in vivo. Nevertheless, these approaches may require specialized experimental procedures, can introduce sequence-dependent biases, and result in incomplete conversion or reduced sensitivity [135,136].

More recently, nanopore-based direct RNA sequencing has ushered in a new era, enabling exploration of the epitranscriptome at unprecedented resolution and enabling m^5^C and m^6^A mapping at single-molecule resolution, without chemical conversion or immunoprecipitation. Direct RNA sequencing also has the potential to provide transcript- and isoform-level information, which may help resolve modification patterns that are difficult to capture using short-read sequencing [132,137,138]. However, current direct RNA sequencing approaches remain limited by sequencing accuracy, variability in false-positive rates, uncertainty regarding appropriate thresholds for modification calling, limited independent validation of model performance, and a lack of consensus on standardized analytical pipelines [137,139]. Collectively, a more comprehensive and accurate assessment of m^6^A and m^5^C landscapes in male reproductive tissues and cells is needed to better define the roles of these RNA modifications in male infertility.

### 4.5. Therapeutic Implications and Challenges

Evidence from genetically modified animal models has demonstrated important roles for m^6^A and m^5^C regulators in spermatogenesis and sperm function, suggesting that m^6^A and m^5^C regulatory pathways may be potential therapeutic targets for male infertility. Therefore, therapeutic targeting of RNA modification machinery and its downstream pathways may offer opportunities to alleviate aberrant RNA regulatory programs and improve cellular dysfunction associated with spermatogenesis and sperm function. However, therapeutic targeting of these pathways remains in its early stages, and few drugs have been tested in preclinical models. Significant challenges remain for clinical application, including achieving sufficient target specificity, minimizing off-target effects, and controlling the magnitude and duration of RNA modification changes. In addition, critical challenges must be addressed before clinical application can be considered, including ensuring sufficient target specificity, minimizing off-target effects, and controlling the magnitude and duration of RNA modifications. Moreover, the potential effects on germ cells and the integrity of the male germline require particular consideration, given the possibility of long-term consequences for reproductive health and offspring. Delivery to the appropriate reproductive tissues or cell populations, treatment timing, dose optimization, and potential toxicity are additional challenges. Therefore, further studies are required to establish specificity, safety, and long-term reproductive consequences before these strategies can be translated into clinical practice.

## 5. Conclusions

Although key parameters, such as sperm concentration, motility, and morphology, are useful for identifying severe conditions such as azoospermia and globozoospermia, they offer limited insight into sperm function or the likelihood of successful fertilization and pregnancy [140]. Importantly, the role of RNA modifications in these processes in male infertility remains unclear. Characterizing the epitranscriptomic landscape of sperm across multiple biological steps may help bridge this gap by revealing molecular pathways that influence sperm competence, fertilization, and early embryonic development.

A major limitation in the clinical management of male infertility is the lack of reliable methods to detect functional defects in sperm. In male infertility, expression levels of regulatory proteins involved in RNA modification and their targets are altered, suggesting that manipulating these regulatory pathways may improve spermatogenesis, enhance sperm quality, or restore reproductive function in selected patients. As profiling and epitranscriptomic integration into clinical practice advance, both the diagnosis and treatment of male infertility can be improved, supporting the development of more individualized reproductive medicine.

## Figures and Tables

**Figure 1:**
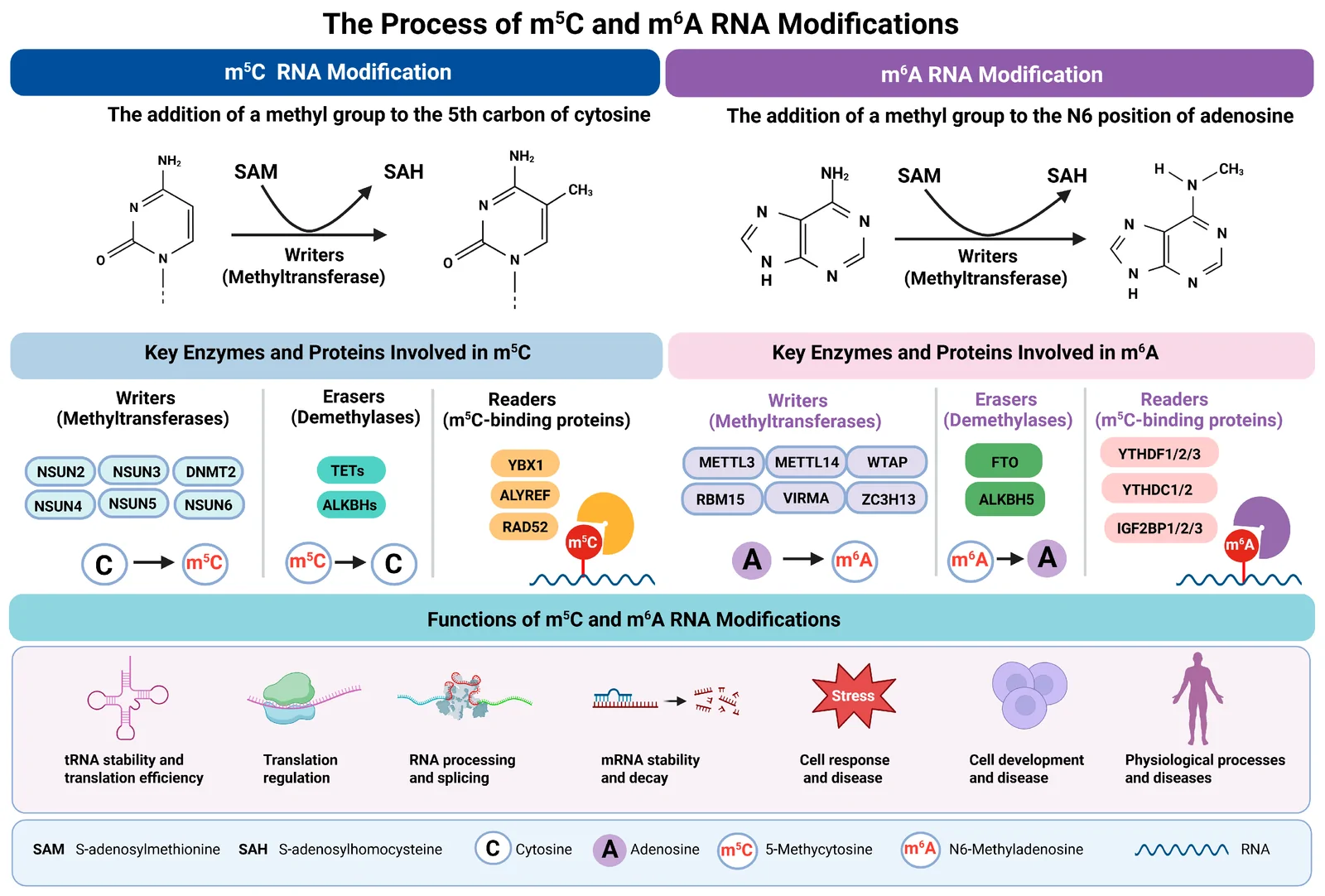
Overview of the mechanisms and biological functions of m^5^C and m^6^A RNA modifications. The top-left panel illustrates m^5^C deposition by m^5^C RNA methyltransferases (writers), whereas the top-right panel depicts m^6^A installation by m^6^A writers. The middle-left and middle-right panels show the diverse regulators involved in m^5^C and m^6^A RNA modification, respectively. The lower panel shows the functional roles of m^5^C and m^6^A RNA modifications in cellular and molecular processes. These epitranscriptomic pathways regulate multiple aspects of RNA metabolism and thereby influence gene expression programs involved in cellular homeostasis, development, differentiation, stress adaptation, and disease pathogenesis, including male infertility.

**Figure 2:**
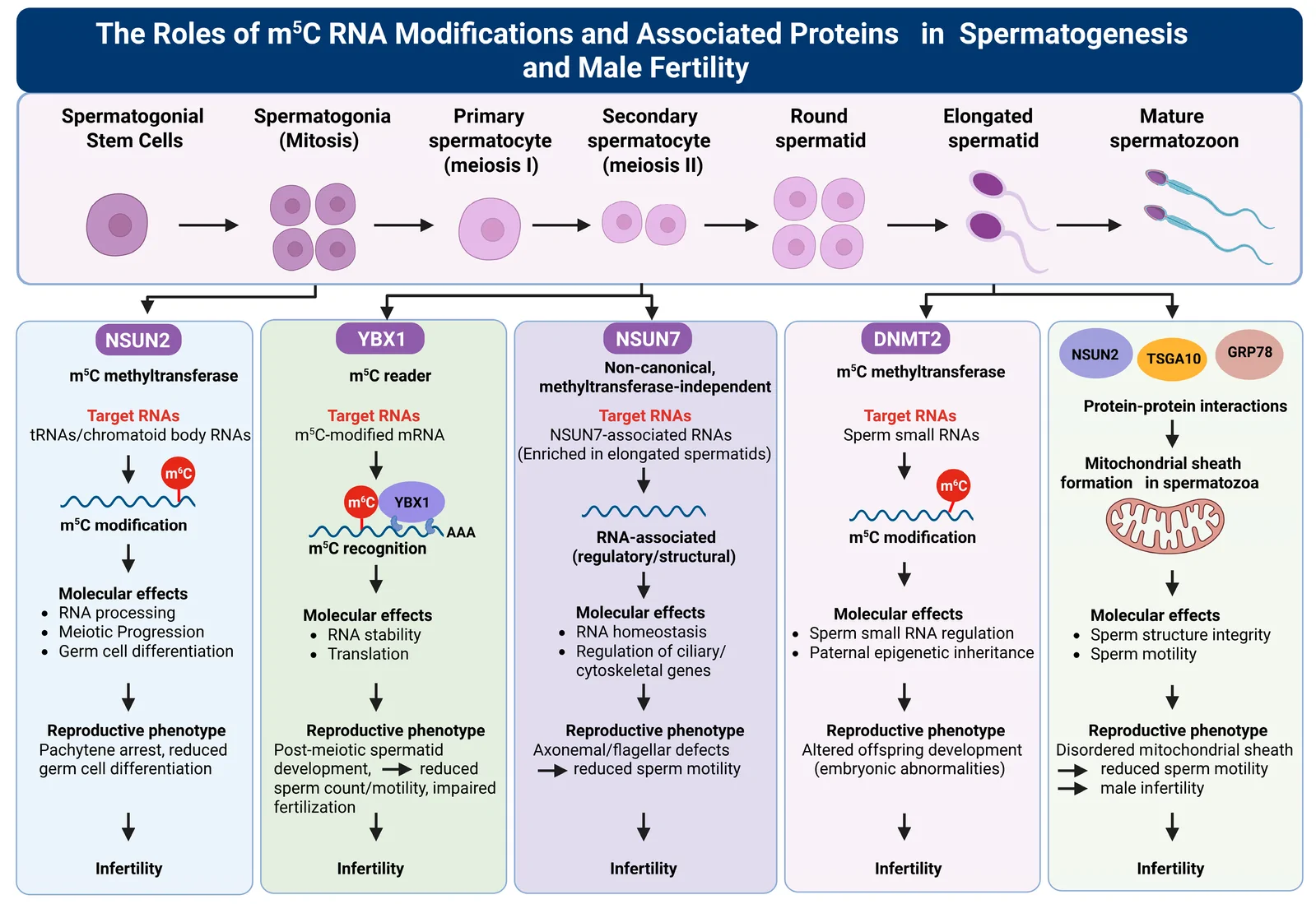
Roles of m^5^C RNA modifications and associated proteins during spermatogenesis and male fertility. The top panel shows a developmental program in which SSCs sequentially differentiate into spermatogonia, primary and secondary spermatocytes, round spermatids, elongated spermatids, and ultimately mature spermatozoa. The lower panel shows the important role of m5C regulatory proteins, including NSUN2, NSUN7, and YBX1, which contribute to multiple stages of this process. Loss of the m^5^C writer, *Nsun2*, results in defective germ cell differentiation and male infertility. NUS7 regulates spermatogenesis through a non-canonical, methyltransferase-independent mechanism. In addition, deficiency of TSGA10, which can interact with NSUN2, leads to abnormal mitochondrial sheath organization, reduced sperm motility, and compromised male fertility. In addition to m^5^C writers, YBX1, as an m^5^C reader, impairs spermatid maturation, and its deficiency leads to fewer elongated spermatids, reduced sperm number and motility, and diminished fertilization capacity. These findings demonstrate that m^5^C-associated proteins regulate spermatogenesis, which is essential for maintaining normal sperm function and male reproductive capacity.

**Figure 3:**
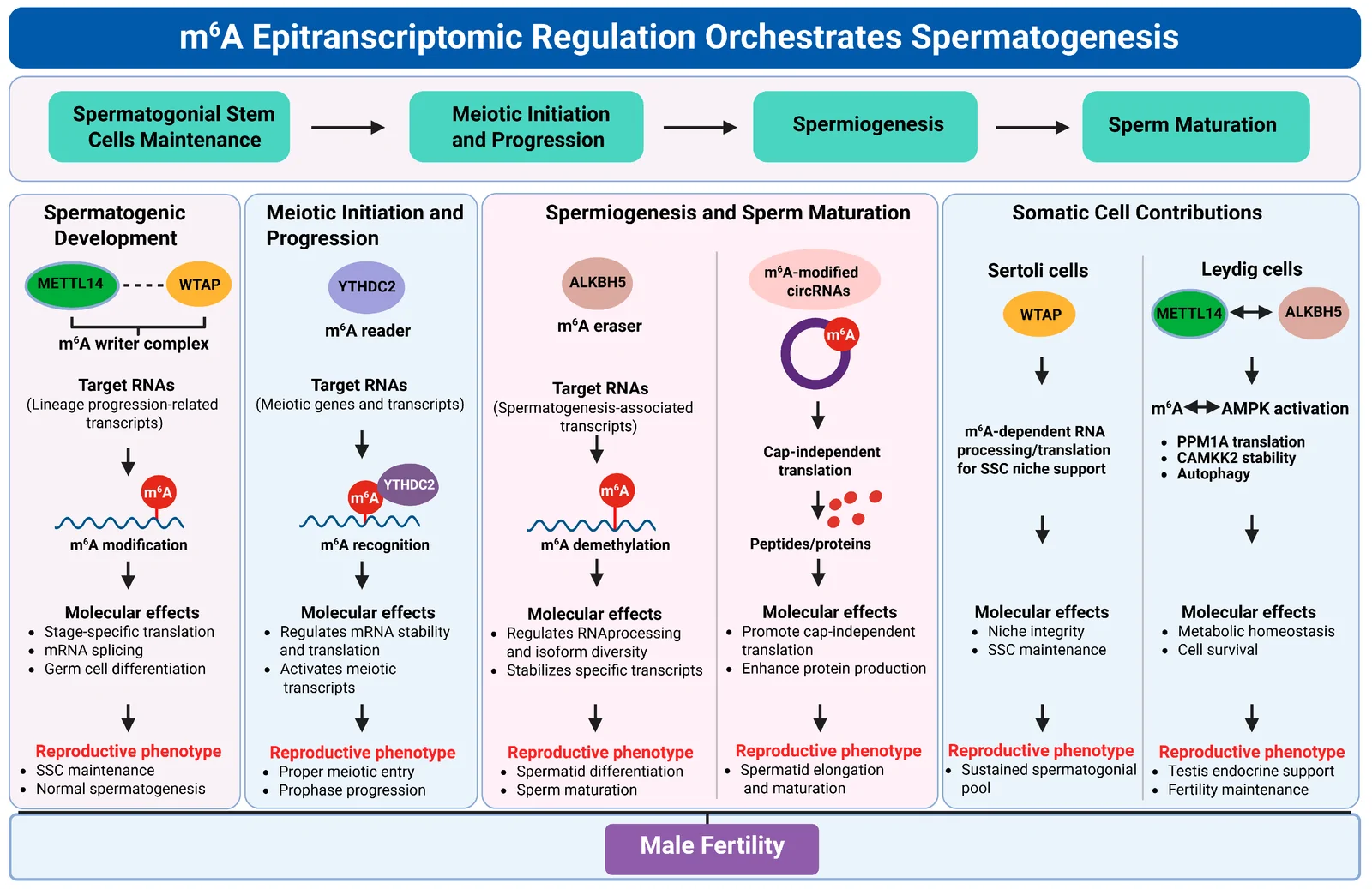
Epitranscriptomic regulation of spermatogenesis and sperm maturation by m^6^A RNA modifications. This schematic summarizes the stage-specific functions of m^6^A RNA modifications during male germ cell development and highlights how epitranscriptomic regulation coordinates spermatogenesis, sperm maturation, and testicular function. The m^6^A methyltransferase machinery, including METTL14 and WTAP, is involved in SSC maintenance and differentiation, while ALKBH5 contributes to spermiogenesis and sperm maturation. The m^6^A reader YTHDC2 is involved in meiotic initiation and progression. Together, these m^6^A regulators coordinate SSC maintenance, meiotic progression, spermiogenesis, sperm maturation, and endocrine function, ultimately supporting normal male fertility.

**Table 1:** The Role of m^5^C in Male Infertility.

Disease	Sample Type	Approach	Mechanism	Biological Effect	Publication Date	Ref.
Male Infertility	*Nsun7* knockout mice, focusing on elongated spermatids and sperm flagella	Genetic inactivation of *Nsun7*, combined with RNA-binding and post-transcriptional analyses to identify NSUN7 RNA targets and assess effects on sperm ultrastructure and motility	NSUN7 binds RNAs in elongated spermatids to regulate post-transcriptional expression; its loss upregulates these RNAs and mispositions longitudinal columns, impairing sperm flagella	*Nsun7* deficiency disrupts post-transcriptional regulation of target RNAs and sperm flagellar organization, resulting in impaired sperm motility and male infertility	April 2025	[63]
Male infertility, spermatogenesis	Genetically engineered mouse model (*Nsun7*^C382A^ knock-in mice), Testis and sperm cells	CRISPR/Cas9-mediated mutagenesis to substitute the putative catalytic cysteine in NSUN7, followed by comprehensive phenotypic analyses	NSUN7 has an atypical catalytic site and regulates spermatogenesis through a non-canonical, methyltransferase-independent mechanism	The catalytic mutant mice exhibit normal fertility, sperm motility, and proper longitudinal column organization in sperm flagella, demonstrating that NSUN7 retains its biological role in spermatogenesis even in the absence of its predicted catalytic activity.	September 2025	[66]
Male Infertility	Adult mouse testes, elongated spermatids, epididymal spermatozoa, haploid spermatids, testicular RNA fractions, purified mouse and human NSUN7 proteins, cultured cells	CRISPR-Cas9 knockout mouse model, UPLC-MS/MS, ultrafast bisulfite sequencing, sequence alignment, AlphaFold structural prediction, ITC assays, site-directed mutagenesis, fertility testing, IVF, CASA sperm analysis, TEM, JC-1 staining, ROS assays, scRNA-seq, DIA proteomics, RNase A treatment assays	*NSUN7* expression is increased in elongated spermatid flagella but lacks RNA m^5^C methyltransferase activity due to impaired SAM binding. Nevertheless, it is required for normal sperm development, as *Nsun7* deficiency causes axonemal and flagellar defects, impaired sperm function, and reduced expression of genes involved in cilium organization and cytoskeletal regulation.	*Nsun7* deficiency causes severely impaired sperm progressive motility, defective sperm flagellar organization, reduced fertilization competence, and complete male sterility without affecting spermatogenesis, sperm count, or mitochondrial function.	December 2025	[64]
Male infertility	*Nsun2* knockout mouse testes, including germ cells at different stages of spermatogenesis	Genetic knockout of *Nsun2* (and combined *Nsun2*/*Dnmt2* deletion), followed by molecular and histological analyses to assess germ cell development, meiotic progression, and expression of RNA-processing factors	NSUN2 in the chromatoid body, controls post-transcriptional RNA processing and tRNA methylation, and its loss impairs meiotic progression	*Nsun2* deficiency results in blocked meiosis at the pachytene stage, reduced germ cell differentiation, and impaired spermatogenesis, ultimately causing male infertility	April 2013	[65]
Male subfertility/infertility	*Ybx1* conditional knockout (cKO) mouse model, analysis of testis and epididymal sperm	Generated a conditional knockout of *Ybx1* and assessment of sperm count, motility, testicular morphology, and fertilization capacity	Regulates post-meiotic spermatid development, via post-transcriptional and m^5^C-dependent epitranscriptomic control of target mRNAs	Loss of *YBX1* leads to reduced sperm count and motility, decreased elongated spermatids, and impaired fertilization capacity, resulting in male subfertility despite normal meiotic progression	August 2025	[67]
Male infertility	*Tsga10*+/- mice generated via CRISPR/Cas9, including analysis of testicular tissue and spermatozoa.	Genetic knockout of *Tsga10*, assess TSGA10 function, sperm morphology, motility, and protein interactions	TSGA10 is essential for sperm mitochondrial sheath formation and interacts with GRP78 and NSUN2, implicating roles in mitochondrial organization, post-transcriptional regulation, and RNA modification–related pathways.	Loss of *Tsga10* leads to male infertility, reduced sperm motility, and disordered mitochondrial sheath formation, highlighting its essential role in sperm structural integrity and reproductive function.	January 2021	[68]
Male reproductive development	Testicular tissue from 9 healthy Simmental bulls at three developmental stages	Direct RNA sequencing (DRS); differential gene expression analysis; alternative splicing analysis; RNA modification association analysis (m^5^C and m^6^A)	m^5^C and m^6^A modulate transcript processing and expression, with m^5^C-associated splicing enhancing isoform-level expression of spermatogenesis-related genes such as MAEL	Identification of developmentally regulated genes linked to testicular growth; enhanced MAEL isoform expression associated with m^5^C-mediated splicing; overall promotion of spermatogenesis and testicular maturation	January 2023	[69]
Sperm development	Testis and epididymis tissues from roosters at different developmental stages	Whole transcriptome sequencing of mRNAs, lncRNAs, and miRNAs, followed by differential expression analysis and construction of ceRNA regulatory networks.	Sperm development is regulated by ceRNA networks affecting MAPK, TGF-β, mTOR, and calcium pathways; although not directly measured, involvement of YBX1 suggests a potential role for m^5^C-mediated epitranscriptomic regulation.	These regulatory networks promote spermatogenesis, sperm maturation, motility, and immune regulation in the epididymis, ultimately supporting sperm viability and reproductive efficiency, potentially through coordinated transcriptional and epitranscriptomic mechanisms	November 2024	[70]
Maternal drug-induced developmental defects	Adult medaka fish sperm and their embryos, with analyses of sperm small RNAs and RNA modifications	Male fish were exposed to methotrexate, followed by small non-coding RNA sequencing, RNA methylation analysis, and injection of sperm RNAs into fertilized eggs to evaluate effects on offspring development.	Paternal MTX exposure alters sperm tRNAs and 5′ tRNA halves and increases m^5^C modifications via DNMT2, suggesting an epitranscriptomic role in developmental programming.	Altered sperm sncRNAs and RNA modifications from MTX-treated males cause cranial cartilage defects in offspring, demonstrating that paternal RNA-mediated epigenetic changes can influence embryonic development	March 2023	[71]
Male infertility	Busulfan-induced mouse model of oligoasthenospermia, testicular tissue and sperm samples	Established the disease model via busulfan treatment and subsequently administered 5- m^5^C levels, followed by evaluation of testicular morphology, sperm parameters, hormone levels, oxidative stress markers, RNA sequencing, and gene expression analyses	m^5^C supplementation restores reduced m^5^C levels in oligoasthenospermia and promotes spermatogenesis through multiple mechanisms	m^5^C upregulation improves testicular morphology, increases sperm concentration and motility, enhances ciliary and cytoskeletal structure, normalizes hormone levels, and reduces oxidative damage, ultimately promoting recovery of spermatogenesis and male fertility	March 2024	[72]

**Table 2:** The Role of m^6^A in Male Infertility.

Disease	Sample Type	Approach	Mechanism	Biological Effect	Publication Date	Ref.
Male Infertility	Mouse spermatogenic cells across five developmental stages	m^6^A methylome profiling, germ cell-specific conditional knockout of *Mettl3* or *Mettl14* using Vasa-Cre and Stra8-GFPCre, translational analysis of spermatogenic transcripts	METTL3/METTL14-mediated m^6^A modification regulates SSC proliferation, differentiation, and spermiogenesis. Loss of m^6^A dysregulates translation of key SSC- and haploid-specific genes essential for germline development.	Germ cell-specific deletion of *Mettl3* or *Mettl14* resulted in loss of m^6^A and depletion of SSCs. Combined deletion of *Mettl3* and *Mettl14* disrupted spermiogenesis and impaired translation of haploid-specific genes, resulting in defective sperm development and infertility-related phenotypes.	October 2017	[13]
Male Infertility	Mouse testes, purified testicular cells/seminiferous tubule cells, spermatozoa, HeLa cells, and mRNA samples	Generation of *Alkbh*5 knockout mice; LC-MS/MS quantification of m^6^A; RNA-seq transcriptomics; RNA immunoprecipitation; in vitro demethylation assays	*ALKBH5* loss increases m^6^A levels, disrupts RNA processing and splicing, and activates p53-associated apoptosis in germ cells.	*ALKBH5* deficiency reduced sperm count and motility, caused abnormal sperm morphology and defective spermiogenesis, and ultimately compromised male fertility	January 2013	[77]
Male infertility	Mouse Sertoli cells; Sertoli cell-specific Wtap conditional knockout mice; testes and SSC niche-associated transcripts	Conditional knockout of *Wtap* in Sertoli cells; MeRIP-seq; ribosome nascent-chain complex-bound mRNA sequencing (RNC-seq); phenotypic analysis of spermatogenesis and fertility	Loss of *Wta*p disrupts m^6^A deposition, leading to abnormal alternative splicing and dysregulated translation of mRNAs encoding SSC niche factors.	Sertoli cell-specific deletion of *Wtap* causes male sterility, progressive depletion of the SSC population, impaired SSC niche function, and defective spermatogenesis.	October 2020	[73]
Male Infertility	Mouse testes and germ cells at different spermatogenic stages (P8, P12, P20); spermatogonia, spermatocytes, and post-meiotic germ cells	m^6^A transcriptome mapping; RNA-seq of wild-type and *Ythdc2* knockout testes; high-throughput m^6^A site mapping; biochemical assays of YTHDC2 helicase/ATPase activity; protein interaction studies	YTHDC2 binds m^6^A- transcripts and promotes their decay via XRN1, while also acting as an RNA helicase/ATPase. Loss of *Ythdc2* causes accumulation of m^6^A-enriched transcripts and failure to suppress mitotic genes, blocking the mitosis-to-meiosis transition.	Arrest of meiosis and impaired spermatogenic differentiation; accumulation of aberrantly expressed germ cell transcripts; failure of germ cells to properly enter meiosis, leading to defective gametogenesis and infertility in mice	October 2017	[74]
Male infertility	*Ythdc2*-deficient mouse male germ cells, including spermatogonia and meiotic germ cells (spermatocytes) from mouse testes	*Ythdc2* knockout mice were analyzed for germ-cell differentiation, meiotic progression, mitotic and meiotic marker expression, YTHDC2 target transcripts, and interactions with piRNA precursors and RNA granule components	Loss of *Ythdc2* disrupts suppression of mitotic genes and activation of meiotic programs, resulting in a mixed mitotic–meiotic cell identity	Germ cells initiate meiosis but fail to progress through meiotic prophase, showing persistent mitotic gene expression, reduced meiotic markers, abnormal mitotic-like divisions, increased apoptosis, defective spermatocyte development, and severe spermatogenic failure leading to male infertility	October 2017	[75]
Male infertility	Mouse pachytene spermatocytes; *Ythdc2*-deficient germ cells; testes from conditional knockout mice	Conditional deletion of *Ythdc2*; YTHDC2-RNA target identification; cytological analysis of meiotic chromosomes, telomere dynamics, microtubule organization, and apoptosis	YTHDC2 maintains the meiotic transcriptome by promoting target mRNA degradation. Its loss leads to accumulation of mitotic transcripts and reduction in meiotic transcripts, largely through m^6^A-independent RNA turnover mechanisms	YTHDC2 deficiency induces aberrant microtubule-dependent telomere clustering, transcriptome reprogramming, pachytene-stage apoptosis, meiotic arrest, and failure of spermatogenesis.	December 2021	[76]
Male Infertility	Mouse spermatogonia cells (*Ythdf2*-knockout spermatogonia)	CRISPR/Cas9-mediated *Ythdf2* knockout, MeRIP-PCR, functional rescue experiments using MMP13 inhibition	YTHDF2, regulates expression of MMPs. Mmp3, Mmp13, Adamts1, and Adamts9 transcripts contain m^6^A modifications and directly interact with YTHDF2. Loss of YTHDF2 disrupts m^6^A-mediated degradation and regulation of these ECM genes.	*Ythdf2* depletion impaired cell–matrix adhesion and reduced proliferation in spermatogonia. Inhibition of MMP13 partially rescued the abnormal phenotypes caused by *Ythdf2* knockout.	January 2020	[78]
Male Infertility	Pachytene spermatocytes and round spermatids isolated from wild-type and Alkbh5 knockout mice	MeRIP-seq, integrated methylome-transcriptome analysis, pathway enrichment analysis, and qPCR validation	Loss of *Alkbh5* increases global m^6^A methylation, alters m^6^A peak distribution and m^6^A-regulated gene expression in germ cells. ALKBH5-mediated m^6^A demethylation regulates post-transcriptional gene expression during germ cell development.	*Alkbh5* deficiency alters transcript abundance and m^6^A methylation patterns in pachytene spermatocytes and round spermatids, leading to impaired germ cell development and male infertility.	March 2022	[79]
Male Infertility	Mouse testes and post-meiotic spermatids	Genetic knockout, m^6^A reader profiling, RNA target analysis, protein interaction studies, and siRNA rescue experiments were used to define IGF2BP3 function in spermiogenesis	IGF2BP3 recognizes m^6^A-modified mRNAs and, through interaction with YBX2, represses translation of target genes such as *Dot1l* and *Hdac11*, regulating histone-to-protamine replacement during sperm maturation	Loss of *IGF2BP3* causes abnormal sperm chromatin remodeling, defective spermatid development, and impaired male fertility; suppression of *Dot1l/Hdac11* rescues sperm defects	January 2026	[80]
Male germ cell development	Murine male germ cells, including late pachytene spermatocytes, round spermatids, elongating spermatids, and mature spermatozoa	circRNA profiling; m^6^A mapping analysis; RNA sequencing; bioinformatic prediction of coding potential; liquid chromatography–mass spectrometry (LC-MS/MS) for peptide identification	m^6^A modification promotes circRNA biogenesis and translation by favoring back-splicing at m^6^A-enriched sites near start and stop codons, generating circRNAs with large ORFs and m^6^A-modified start codons that enable cap-independent protein translation.	m^6^A promotes production and translation of coding circRNAs during spermiogenesis, sustaining protein synthesis in male germ cells. Hundreds of circRNA-derived peptides were detected, revealing a novel role of m^6^A in circRNA biogenesis and translation	March 2020	[81]
Male Infertility	Human sperm from 49 participants (13 healthy controls, 21 AZS patients, 15 TZS patients); total sperm RNA and size-fractionated RNA fragments (>80 nt and other fractions)	LC-MS/MS; comparative cohort analysis; correlation analysis with clinical parameters	Altered sperm RNA modification profiles, with multiple modification types (13 in total RNA and 16 in fragments), show coordinated changes in m^5^C, m^6^A, and m^1^A that correlate with sperm motility, linking RNA modification signatures to sperm function and quality	AZS and TZS reduced sperm motility; identification of RNA modification signatures as potential biomarkers for sperm quality and male infertility assessment	August 2022	[82]
Reproductive model	Bull spermatozoa collected from MSTN-edited bulls and wild-type control bulls	m^6^A-seq, RNA-seq, bioinformatics analysis	MSTN editing reshapes the sperm m^6^A landscape, affecting transcripts involved in GPCR signaling, membrane organization, and cytoskeletal regulation. The RHOA-centered network suggests that m^6^A modulates sperm function by regulating cytoskeleton and membrane-related genes.	MSTN editing induced extensive remodeling of the sperm m^6^A epitranscriptome, affecting genes involved in pathways related to sperm structure and function	January 2025	[83]
Asthenozoospermia	Human semen/sperm RNA samples (20 asthenozoospermia patients, 32 healthy controls)	LC-ESI-MS/MS for RNA m^6^A quantification; real-time PCR analysis of m^6^A regulators (METTL3, METTL14, WTAP, FTO, ALKBH5, YTHDF2)	Increased m^6^A levels associated with elevated expression of METTL3 and METTL14; METTL3 positively correlated with m^6^A content	Elevated m^6^A content was identified as a risk factor for asthenozoospermia; increased m^6^A negatively correlated with sperm progressive/non-progressive motility and positively correlated with sperm immotility	April 2016	[84]
Male Infertility	Human spermatozoa from male partners of couples with unexplained recurrent pregnancy loss (URPL) and fertile controls; zebrafish embryos	MeRIP-seq; integrated multi-omics analyses (transcriptomics, proteomics, metabolomics); zebrafish embryo functional studies	Aberrant sperm m^6^A RNA methylation altered expression and methylation status of SEMA5A, MT-ATP6, ZNF662, and KDM4C. These dysregulated paternal epigenetic signals disrupted key embryonic signaling pathways and zygotic genome activation during embryogenesis.	Increased embryonic mortality and developmental malformations in zebrafish embryos; impaired embryogenesis potentially contributing to recurrent pregnancy loss; identified genes may serve as diagnostic biomarkers for paternal-factor-associated URPL	March 2025	[85]
Male infertility	Prepubertal Sprague–Dawley rat testes exposed to DEHP, followed by analyses of histology, oxidative stress, apoptosis, and m^6^A-related changes	Oral DEHP exposure in rats followed by histological, hormonal, oxidative stress, apoptosis, and global m^6^A analyses, including assessment of *FTO/YTHDC2* expression and MeRIP-qPCR evaluation of *Nrf2* mRNA methylation	DEHP exposure alters FTO and YTHDC2 expression, increases global m^6^A methylation and enhances m^6^A modification of *Nrf2* mRNA, and impairs Nrf2-mediated antioxidant signaling, leading to oxidative stress and testicular damage	DEHP exposure reduced testosterone, altered spermatogenesis gene expression, increased oxidative stress, and elevated germ-cell apoptosis. These changes lead to impaired spermatogenesis and male reproductive dysfunction.	April 2020	[86]
Male Infertility	Human semen samples and testicular tissue from 77 men undergoing infertility evaluation	MiSeq of *FTO* and *ALKBH5* genes; variant calling with GATK Unified Genotyper; immunohistochemistry and Western blot for *ALKBH5/FTO* expression in testis	Genetic variants and missense mutations in *FTO* (including p.Cys326Ser and p.Ser256Asn) may impair RNA demethylation function.	FTO genetic variants were associated with reduced semen quality, suggesting dysfunctional m^6^A RNA demethylation contributes to impaired male fertility	May 2016	[87]
Male Infertility	Varicocele rat testes; GC-2 spermatocyte cells under oxidative stress	MeRIP-seq, MeRIP-qPCR, RIP-qPCR, CRISPR/dCas13b-ALKBH5-mediated targeted demethylation	Varicocele-induced oxidative stress increases m^6^A levels and *PLOD2* expression, with IGF2BP2 stabilizing PLOD2 mRNA via its m^6^A-modified 3′-UTR. CRISPR/dCas13b-ALKBH5–mediated demethylation reduces PLOD2 m^6^A levels and expression.	Elevated PLOD2 contributes to oxidative stress-related spermatogenic dysfunction. Demethylation of *PLOD2* promoted GC-2 cell proliferation and reduced apoptosis under oxidative stress, suggesting therapeutic potential for male infertility.	September 2023	[88]
Male Infertility	Mouse testes, spermatocytes, oocytes, HeLa cells, adult and 8.5–16.5 d.p.p. mouse testes	CRISPR/Cas9-mediated *Ythdc2* knockout mice, RIP-seq, CLIP-seq, m^6^A-seq, ribosome profiling, polysome profiling, tethered luciferase reporter assay, tandem affinity purification-mass spectrometry	YTHDC2 binds GGACU motifs near stop codons, promoting translation while facilitating target mRNA decay through factors such as XRN1, UPF1, and MOV10. Loss of *Ythdc2* disrupts regulation of meiotic and spermatogenesis-related transcripts, including *Smc3* and *Cep76*.	*Ythdc2* knockout mice are infertile with smaller testes and ovaries, absence of spermatozoa, meiotic arrest at the zygotene-like stage, failure to progress to pachytene, increased germ-cell apoptosis, reduced spermatocyte numbers, defective spermatogenesis, and impaired germ-cell development.	September 2017	[89]
Abnormal testis development	Testis tissues from Xenopus laevis (African clawed frog), including control and 100 μg/L atrazine-exposed animals.	Transcriptome-wide m^6^A-seq (MeRIP-seq) comparing AZ-treated and control testes, including differential m^6^A peak and mRNA correlation analyses, followed by KEGG pathway enrichment analysis	m^6^A is enriched near start and stop codons in *X. laevis*. Atrazine exposure alters the testicular m^6^A epitranscriptome, generating 1380 differentially methylated sites linked to immune, cell junction, and lipid metabolism pathways, with m^6^A positively correlating with mRNA expression during testicular development.	Atrazine-induced changes in the testicular m^6^A methylome disrupt pathways involved in testis development, with dysregulation of cell junction, immune, and lipid metabolism contributing to impaired reproductive function, highlighting an epitranscriptomic mechanism of environmental toxicity	April 2020	[90]
Male infertility	Mouse germ cells and testes (P6, P10, P12, adult); PLZF-positive spermatogonia; spermatocytes at different meiotic stages	Germ cell-specific *Mettl3* knockout using Vasa-Cre/loxP, combined with immunostaining, TUNEL assays, RNA-seq, m^6^A profiling (miCLIP-seq), alternative splicing analysis, and m^6^A-RIP-qPCR validation	Loss of *Mettl3* disrupts m^6^A methylation, causing abnormal splicing of key germline transcripts, reduced expression of differentiation and meiotic genes, and meiotic failure due to defective DNA repair and synapsis.	Severe male infertility phenotype characterized by reduced testis size, loss of germ cell differentiation, arrest at zygotene stage of meiosis (no pachytene formation), increased germ cell apoptosis, and complete infertility in mice	September 2017	[91]
Male Infertility	Mouse testes; purified pachytene spermatocytes, round spermatids, elongating spermatids; epididymal sperm	*Alkbh5* knockout mice, RNA-seq, m^6^A-RIP-seq, immunofluorescence, TUNEL assay, histology, qPCR, sperm analysis	ALKBH5 is a nuclear m^6^A demethylase that regulates pre-mRNA splicing and long 3′-UTR transcript formation in spermatocytes and spermatids. Its loss increases m^6^A retention, causing abnormal splicing, shorter isoforms, and enhanced mRNA degradation during spermiogenesis	Defective meiotic progression and spermiogenesis, increased germ-cell apoptosis, depletion of pachytene spermatocytes and spermatids, abnormal sperm morphology and motility, oligoasthenoteratozoospermia-like phenotype, male infertility	January 2018	[92]
Idiopathic azoospermia	Public human idiopathic azoospermia (IA) transcriptomic datasets/testicular gene expression data	Bioinformatics analysis integrating sequencing data and public datasets; pathway and gene screening analysis	Disrupted RNA metabolism and abnormal m^6^A regulation in spermatogenic cells; defects in ciliary structure and function proposed as key pathogenic mechanisms underlying spermatogenic failure	Impaired spermatogenesis leading to absence of healthy sperm and infertility; identification of candidate key genes and potential therapeutic targets/drugs for IA	July 2020	[93]
Azoospermia/Oligospermia	Leydig cells (LCs), including stem Leydig cells and adult Leydig cells; HsCG-treated LC models	Analysis of m^6^A regulators during LC differentiation; HsCG stimulation experiments; mechanistic studies of m^6^A-mediated RNA regulation; transcription factor/promoter interaction studies (CEBPB, TFEB); autophagy and AMPK pathway analyses	Decreased METTL14 and increased ALKBH5 reduce global m^6^A during Leydig cell differentiation and hCG stimulation, promoting AMPK-driven autophagy via reduced PPM1A translation and increased CAMKK2 stability. ALKBH5 is upregulated by CEBPB/TFEB, while hCG destabilizes METTL14	Reduced m^6^A methylation promoted autophagy and supported testosterone synthesis in Leydig cells; dysregulation of this pathway may contribute to impaired testosterone production associated with azoospermia and oligospermia, suggesting m^6^A regulators as potential therapeutic targets	February 2021	[94]
Oligoasthenoteratozoospermia	Mouse germ cells and testes (C57BL/6J background); sorted spermatogenic cell populations (diplotene spermatocytes, round spermatids via FACS)	Conditional knockout of *Ythdf2* in germ cells; MeRIP-qPCR validation of m^6^A targets; integration with single-cell RNA-seq datasets;	Deficiency of *Ythdf2* impairs degradation of m^6^A-modified transcripts, causing their accumulation and disrupting transcript turnover required for germ cell differentiation during spermatogenesis	Oligoasthenoteratozoospermia phenotype characterized by abnormal sperm count, motility, and morphology; increased germ cell apoptosis; disrupted progression of spermatogenesis and impaired fertility in mice	December 2021	[95]
Male reproductive function	Mouse-derived GC-1 spermatogonia cell line	Melatonin treatment combined with VIRMA knockdown and overexpression; m^6^A dot blot analysis, and RNA sequencing; PI3K inhibitor (LY294002) validation	Melatonin upregulates the VIRMA expression, increasing global m^6^A methylation and activating PI3K/AKT signaling in spermatogonia. Silencing or overexpression of Virma can reverse these effects.	Melatonin promotes spermatogonia viability and proliferation while suppressing apoptosis. Increased VIRMA-dependent m^6^A methylation enhances spermatogonial activity and may contribute to improved spermatogenesis through activation of PI3K/AKT signaling.	December 2022	[96]
Male Infertility	Mouse testes and sperm from multigenerational high-fat diet models; human sperm samples from overweight/obese donors	Multigenerational high-fat diet mouse model; cadmium exposure studies; retinoic acid measurements; gene/m^6^A expression analyses; AAV9-Wt1 rescue experiments; METTL3 inhibitor (STM2457) treatment; case–control human sperm analysis	Paternal obesity increased METTL3-mediated m^6^A modification of Wt1, suppressing retinoic acid synthesis and impairing spermatogenesis. METTL3 inhibition reduced Wt1 m^6^A levels and rescued reproductive defects	Progressive decline in spermatogenesis, retinoic acid levels, and sperm count across generations exposed to high-fat diet; increased susceptibility to cadmium-induced spermatogenic disorders; overweight/obese human donors showed elevated sperm m^6^A levels and reduced sperm concentration	February 2024	[97]
Male infertility.	Mouse spermatogonia (likely GC-1 spg spermatogonial cell line) with CRISPR/Cas9-mediated *Fto* depletion.	CRISPR/Cas9 *Fto* knockout with rescue (wild-type and inactive mutants) combined with cell-cycle, chromosome stability, and gene expression analyses of mitotic checkpoint and G2/M regulators, plus identification of direct FTO-regulated m^6^A targets.	*Fto* loss increases m^6^A levels, reducing expression of mitotic checkpoint and G2/M regulators, with effects dependent on its catalytic activity, as only wild-type FTO rescues the phenotype.	*Fto* depletion induces G2/M arrest in spermatogonia, disrupting mitotic checkpoint control and cell-cycle progression, leading to impaired proliferation and male infertility, and highlighting the important role of m^6^A demethylation in germ-cell genomic stability.	January 2019	[98]
Male infertility	Mouse GC-1 spermatogonia-derived germ cells treated with MA2, a selective FTO demethylase inhibitor.	MA2-mediated FTO inhibition was combined with m^6^A/m^6^Am quantification and analyses of proliferation, apoptosis, *Cdk* expression, RNA stability, and m^6^A-dependent *Cdk2* 3′UTR regulation.	FTO maintains the stability of cell-cycle transcripts in spermatogonia. Its inhibition increases m^6^A levels, promoting *Cdk2* mRNA degradation via the *Cdk2* 3′UTR and impairing spermatogonial proliferation.	FTO inhibition increases m^6^A levels, decreases CDK levels, induces G1/S arrest and spermatogonial proliferation, and impaires germ cell maintenance, contributing to defective spermatogenesis and male infertility.	July 2019	[99]

## Data Availability

No new data were created or analyzed in this study. Data sharing is not applicable to this article.

## Abbreviations

The following abbreviations are used in this manuscript:

A-to-I	Adenosine-to-inosine RNA editing
AKT	Protein kinase B signaling pathway
ALKBH5	AlkB homolog 5 RNA demethylase
AMPK	AMP-activated protein kinase
CatSper	Cation Channel of Sperm
CCL20	C-C motif chemokine ligand 20
COC	Cumulus-oocyte complex
circRNA	Circular RNA
ceRNA	Competing endogenous RNA
CRISPR	Clustered regularly interspaced short palindromic repeats
DEFB119	Defensin beta 119
DEHP	Di(2-ethylhexyl) phthalate
DNMT2	DNA methyltransferase 2 (tRNA methyltransferase)
EDC	Endocrine-disrupting chemical
FTO	Fat mass and obesity-associated protein
GRP78	Glucose-regulated protein 78
G2/M	Cell-cycle phase transition
HDAC11	Histone deacetylase 11
HPG	Hypothalamic-pituitary-gonadal
IGF2BP2	Insulin-like growth factor 2 mRNA-binding protein 2
IZUMO1	Izumo sperm-egg fusion protein 1
KDM4C	Lysine demethylase 4C
KEGG	Kyoto Encyclopedia of Genes and Genomes
LC-MS/MS	Liquid chromatography–tandem mass spectrometry
lncRNA	Long non-coding RNA
MAPK	Mitogen-activated protein kinase
METTL3	Methyltransferase-like 3
METTL14	Methyltransferase-like 14
MMPs	Matrix metalloproteinases (MMP3, MMP13)
m^6^A	N6-methyladenosine
m^5^C	5-methylcytosine
m1A	N1-methyladenosine
m7G	N7-methylguanosine
Nrf2	Nuclear factor erythroid 2–related factor 2
NSUN2	NOP2/Sun RNA methyltransferase 2
NSUN7	NOP2/Sun RNA methyltransferase 7
PI3K	Phosphoinositide 3-kinase
piRNA	Piwi-interacting RNA
PRISMA	Preferred Reporting Items for Systematic Reviews and Meta-Analyses
PLOD2	Procollagen-lysine,2-oxoglutarate 5-dioxygenase 2
PUS7	Pseudouridine synthase 7
RHOA	Ras homolog family member A
RNP	Ribonucleoprotein
ROS	Reactive oxygen species
SAM	S-Adenosylmethionine
SSC	Spermatogonial stem cell
scRNA-seq	Single-cell RNA sequencing
SED1	Sperm–Egg Binding Protein 1
Sertoli cells	Testicular supporting somatic cells
SNARE	Soluble N-ethylmaleimide-sensitive factor Attachment Protein Receptor
SRY	Sex-determining region Y
TGF-β	Transforming growth factor beta
TRP	Transient Receptor Potential
TSGA10	Testis-specific gene antigen 10
tsRNA	Transfer RNA-derived small RNA
VIRMA	m^6^A methyltransferase complex regulator
WTAP	Wilms tumor 1-associated protein
YBX1	Y-box binding protein 1
YTHDC2	YTH domain-containing protein 2
YTHDF2	YTH N6-methyladenosine RNA binding protein 2
ZP3	Zona pellucida glycoprotein 3
Ψ	Pseudouridine
